# The Role of Ultrasound Pleural Irregularities in the Identification of Interstitial Lung Disease in Idiopathic Inflammatory Myopathies

**DOI:** 10.3390/jpm16030162

**Published:** 2026-03-14

**Authors:** Linda Carli, Simone Barsotti, Chiara Romei, Andrea Delle Sedie, Federico Fattorini, Michele Diomedi, Elisa Cioffi, Elenia Laurino, Chiara Cardelli, Gaetano La Rocca, Marta Mosca

**Affiliations:** 1 Rheumatology Unit, Department of Medical and Surgical specialties, Azienda Ospedaliero Universitaria Pisana (AOUP), 56126 Pisa, Italy; adellesedie@gmail.com (A.D.S.); elenia.laurino@gmail.com (E.L.); marta.mosca@unipi.it (M.M.); 2 European Reference Network (ERN) ReCONNET, via Roma 67, 56126 Pisa, Italy; Full Member; 3 Rheumatology Unit, Department of Internal Medicine, Ospedale Versilia, 55049 Viareggio, Italy; simone.barsotti.pisa@gmail.com; 4 Department of Translational Radiology, University of Pisa, 56126 Pisa, Italy; chiara.romei@unipi.it; 5 Rheumatology Unit, Department of Clinical and Experimental Medicine, University of Pisa, 56126 Pisa, Italy; federicofattorini19@gmail.com (F.F.); michelediomedi64@gmail.com (M.D.); chiara.cardelli@med.unipi.it (C.C.); gaetano.larocca@phd.unipi.it (G.L.R.); 6 Azienda USL Toscana Centro, 50122 Florence, Italy; eli.cioffi@gmail.com; 7 Department of Medical Biotechnologies, University of Siena, 53100 Siena, Italy

**Keywords:** lung ultrasound, interstitial lung disease, myositis

## Abstract

**Background**: Interstitial lung disease (ILD) is the most frequent extra-muscular manifestation in patients with idiopathic inflammatory myopathies (IIMs). Although high-resolution chest tomography (HRCT) represents the gold standard for the evaluation of ILD, lung ultrasound (LUS) might be a useful tool for its assessment. The aim of our study was to evaluate the number and distribution of pleural irregularities (PIs) identified by lung US in a cohort of patients with IIMs and to find possible correlations with clinical, serological and HRCT data to verify the potential usefulness of lung US for the study of ILD in patients with IIM. **Patients and methods**: Fifty-three patients with IIM according to EULAR/ACR classification criteria were enrolled. All patients underwent a clinical evaluation with measurement of disease activity and myositis-specific autoantibodies, pulmonary function tests, HRCT evaluated with the Warrick score, and lung US for the measurement of PIs. **Results**: The number of PIs was higher in patients with myositis-specific autoantibodies, particularly those with anti-synthetase autoantibodies (*p* < 0.001) and in patients with high-grade dyspnea (*p* < 0.03). A negative correlation was identified between PIs and pulmonary function tests, particularly TLC (r = −0.74; *p* < 0.001) and DLCO (r = −0.56; *p* < 0.001). Interestingly, PI score was higher in patients with ILD identified with HRCT (*p* = 0.015) and a positive correlation between PIs and Warrick score (r = 0.542; *p* < 0.001) was also found. **Conclusions**: The study of PIs with lung US represents a promising diagnostic tool for the bedside evaluation of patients with IIMs. This can possibly allow for a reduction in unnecessary HRCTs, reducing the exposition of patients to ionizing radiations, optimizing resources and reducing the costs of patients’ management.

## 1. Introduction

Idiopathic inflammatory myopathies (IIMs) are a group of systemic autoimmune diseases characterized by an inflammatory process affecting the skeletal muscle. Recently, the term “pneumomyositis” was created, to underline the high prevalence of lung involvement in IIMs [1].

Interstitial lung disease (ILD) represents the most frequent pattern of pulmonary involvement in IIMs and the estimated prevalence of ILD is about 50%, ranging from 21% to 78% in different studies [2,3].

ILD is usually evaluated through pulmonary function tests (PFTs) and, for a better characterization, by high-resolution computed tomography (HRCT).

IIM patients PFTs, combined with an evaluation of the diffusing capacity of the lungs for carbon monoxide (DLCO), usually show the presence of a restrictive pulmonary pattern characterized by a reduction in the total lung capacity (TLC) and in the DLCO. The main limitation of this test, however, is represented by the difficulties that patients with IIMs may have in performing PFTs, due to severe muscular hyposthenia [4].

To date, HRCT represents the gold-standard technique for the diagnosis and the evaluation of ILD [5]. Non-specific interstitial pneumonia (NSIP), usual interstitial pneumonia (UIP), or features of both are the most commonly encountered imaging patterns for ILD in IIM. Consolidated areas are less frequently seen, typically representing organizing pneumonia (OP) or diffuse alveolar damage (DAD) [6].

Although modern CT scanners utilize lower doses of ionizing radiation, multiple scans on patients over time increases risks related to radiation [7] and repeat imaging should only be undertaken when strictly necessary, such as in cases of clinical or functional deterioration.

Therefore, IIM patients could benefit from an alternative technique able to assess inflammatory lung involvement.

Moreover, in the era of personalized medicine, there is a growing need for non-invasive, bedside tools that allow for patient-specific monitoring without the cumulative risks of ionizing radiation.

In recent years, lung ultrasound (LUS) has been proposed as a possible tool for the study of connective tissue disease-associated ILD. Its assessment is based on two different US findings: B-lines (BLs) and pleural line irregularities (PIs) according to the Outcome Measures in Rheumatology (OMERACT) definitions [8].

BLs are defined as vertical hyperechoic reverberation artifacts arising from the pleural line, extending to the bottom of the screen without fading and moving synchronously with lung sliding, while PIs are defined as a loss of regularity that may be associated with an increase in thickness (either focal, diffuse, linear, or nodular) [8]. Despite its advantages—absence of radiation, bedside applicability, and repeatability—LUS has intrinsic physical and technical limitations. Indeed, US cannot penetrate normally aerated lung, limiting the assessment to subpleural regions; deeper parenchymal abnormalities may therefore remain undetected. Moreover, the technique may be influenced by body habitus, chest wall configuration, and coexisting pulmonary conditions.

Particular interest has been placed on the study of BLs as ILD expression in patients affected by systemic sclerosis (SSc) [9,10], but recently lung US has been applied to other autoimmune diseases such as Sjogren Syndrome (SS), Rheumatoid Arthritis (RA), Systemic Lupus Erythematosus (SLE) and Anti-Synthetase Syndrome (ASyS) [9,11,12,13]. As the US evaluation of BLs can be complex and time-consuming for routine clinical practice, the evaluation of PIs has been proposed for patients with SSc, ASyS and SpA [14,15,16].

The diagnosis of ILD is a critical factor in both the therapeutic management and prognosis of patients with IIM. Indeed, the identification of ILD often mandates a more intensive treatment strategy, typically involving induction therapy with high-dose GC followed by cyclophosphamide, mycophenolate mofetil, or rituximab. Consequently, the availability of non-invasive diagnostic tools for early detection is essential to improving the quality of care and clinical outcomes for IIM patients with pulmonary involvement [17].

Furthermore, a tailored diagnostic approach, where LUS could serve as a first-line filter for the use of high-resolution imaging, could serve in optimizing the management of the heterogeneous lung involvement we can observe in IIMs.

The aim of our study was to evaluate the number and distribution of PIs identified by LUS in a cohort of patients with IIMs who underwent a HCRT for clinical reasons; secondly, we searched for correlations with clinical, serological and HRCT data. Finally, we evaluated the reliability of the exam, by estimating the intra- and inter-operator variability.

## 2. Methods

This was a prospective, monocentric observational study performed at the Rheumatology Unit of the University Hospital of Pisa (Italy). Patients were consecutively enrolled between 2018 and 2024 during routine outpatient clinical activity.

### 2.1. Patients

Patients classified with IIM according to EULAR/ACR criteria [18] who had undergone HRCT as usual clinical practice in our center (Table 1) within the two months prior to enrollment were included in the study. Patients were not required to meet all the specified criteria to undergo HRCT; instead, the examination was performed when one or more criteria were present, based on the physician’s clinical judgment. Furthermore, no major therapeutic changes or acute respiratory events occurred in the interval between the HRCT and LUS examinations.

Patients under the age of 18, pregnant or breastfeeding women, patients who did not sign the informed consent form, and those with other cardiopulmonary pathologies and therapies with ACE inhibitors known to cause cough as a side effect were excluded.

Moreover, patients were excluded in the presence of conditions potentially interfering with lung ultrasound findings or ILD evaluation, specifically:

- Cardiac disease and pulmonary congestion: Patients with known heart failure (reduced or preserved ejection fraction), clinically significant valvular disease, or evidence of pulmonary congestion were excluded. Heart failure was ruled out through clinical evaluation, echocardiography when available, and natriuretic peptides (NT-proBNP). However, cardiac congestive failure, while producing BLs, does not produce any PIs.
- Pulmonary arterial hypertension (PAH): Patients with confirmed PAH (right heart catheterization PAPm > 20 mmHg and wedge pressure < 15 mmHg) were excluded.
- Cardiac involvement related to IIM: Patients with clinically myocarditis, perimyocarditis, or arrhythmias were excluded.
- Active pulmonary infections: Patients with recent or ongoing viral or infectious pneumonitis were excluded, including SARS-CoV-2, influenza, CMV, or bacterial pneumonia. Infections were ruled out through clinical assessment, laboratory testing, microbiological/virological testing when indicated and HRCT findings.
- Other pulmonary diseases: Patients with chronic obstructive pulmonary disease (moderate–severe), asthma requiring regular therapy, pulmonary malignancy, idiopathic pulmonary fibrosis unrelated to IIM, pneumothorax, or significant pleural effusion were excluded.
- Connective tissue disease overlap: Patients fulfilling classification criteria for other connective tissue diseases (e.g., systemic sclerosis or mixed connective tissue disease) were excluded. Isolated serological positivity (e.g., anti-PM/Scl) without clinical overlap features was not considered an exclusion criterion.

At enrollment, all patients underwent a clinical rheumatological evaluation, including skin, muscular and pulmonary domains. Pulmonary involvement was evaluated by the presence of chronic cough (defined as a cough that lasts longer than 8 weeks), dyspnea by means of the MRC scale [19], and fine crackles at thoracic auscultation.

Myositis disease activity parameters were collected according to International Myositis Assessment and Clinical Studies (IMACS) criteria [20] for:

- Muscle enzymes such as creatine kinase (CK), transaminases, aldolase, and lactate dehydrogenase (LDH);
- Manual muscle test 8 (MMT8);
- Physician Visual Analog Scale (VAS);
- Patient’s VAS;
- Health Assessment Questionnaire (HAQ);
- Myositis disease activity assessment visual analog scale (MYOACT).

Both myositis-specific and myositis-associated autoantibodies (respectively MSAs and MAAs) were tested, by using line blot (Myositis profile 3, Euroimmun, Lubeck).

Additionally, patients were asked to complete Patient Reported Outcome (PRO) questionnaires about respiratory symptoms: the Leicester Cough Questionnaire (LCQ) [21] and Saint George Respiratory Questionnaire (SGRQ) [22].

For the evaluation of lung involvement, data from PFTs were collected for each patient: forced expiratory volume at first second (FEV1), FEV1/vital capacity (VC), total lung capacity (TLC), DLCO, and DLCO/alveolar volume (AV) as a percentage of the reference values.

### 2.2. Lung US Assessment

Lung US was performed on the anterior and posterior thoracic wall by using an MyLab 25 Gold US machine (Manufacturer ESAOTE, Genoa, Italy) and a 6–18 MHz linear probe. A total of 53 intercostal spaces were scanned:

- Anterior wall: 12 intercostal spaces of the right hemithorax (II, III, IV and V parasternal, emiclavear and anterior axillar) and 9 of the left hemithorax (II, III and IV parasternal, emiclavear and anterior axillar);
- Postero-superior wall: 10 intercostal paravertebral spaces from II to VI (5 left and 5 right) and 6 posterior axillaries (from II to IV—three left and three right);
- Postero-inferior wall: 8 intercostal paravertebral spaces from VII to X (4 left and 4 right) and 8 subscapolaris spaces from VII to X (4 left and 4 right).

Each space was assessed on a longitudinal scan (with respect to the long axis of the ribs) at a frequency of 10 MHz, fixing the focus on the pleural line, to evaluate the pleural profile for PI assessment, assigning a score according to a 3-point Likert scale (regular = 0, mild irregularity = 1, and severe irregularity = 2) [15]. PIs are defined as a loss of regularity that may be associated with an increase in thickness, either focal, diffuse, linear, or nodular [8]. Moderate and severe changes were defined according to Pinal-Fernandez et al. [15]. A score of 0 was assigned to a normal pleural line, a score of 1 was given if pleural irregularity was moderate and a score of 2 if it was severe. To date, no cut-off score has been established for PI positivity and the already published cut-off of 24% from Pinal-Fernandez et al. was related to a sensitivity of 79% and a specificity of 100% for the diagnosis of ILD, in a population of patients with SSc and ASyS, with respect to the HRCT findings.

Each space was summed to obtain the total PI score, ranging from 0 to 106.

LUS still needs standardization and no consensus on the number of intercostal spaces to be assessed exists. According to our experience and the kind of probe we use, we decided to eliminate the mid-axillary line from our evaluation, to avoid duplication (the dimension of the footprint of our probe does not allow us to ensure we do not count part of the space twice while assessing the anterior, mid and posterior axillary line). We therefore decided to consider and evaluate only the PIs present where the pleural line was perpendicular to the ultrasound beam, to avoid blurring and a change in pleural line shape due to physics rather than real changes in the structure.

We assessed the results for both the comprehensive and reduced (only postero-inferior intercostal spaces) scanning protocols, to validate a less time-consuming examination. The reduced scanning protocol was arbitrary decided according to the more frequently involved regions in IIM-related ILD [23].

Finally, LUS cut-offs for the presence of ILD were determined only after considering the HRCT results.

We decided to evaluate only PIs, without vertical artifacts, because while PIs are a well-recognized ultrasound feature of interstitial involvement, B-Lines (BLs) present certain challenges regarding feasibility. Specifically, they require more time for a thorough examination and can be difficult to differentiate from other vertical artifacts, such as Z-lines. In contrast, PIs appear to be more easily evaluated and potentially more specific in this clinical context [24]. Based on these considerations, we a priori selected the evaluation of PIs as the primary objective of this study, aiming to identify a simpler and more feasible marker for routine clinical practice

The exam was performed by a trained rheumatologist (EC) and in a subgroup of patients; thoracic US was repeated by EC two days after the first evaluation (10 patients) and by a second operator (SB) blinded to the results of the first evaluator (14 patients), to estimate the intra- and inter-operator agreement. Both examiners were blinded to clinical findings, HRCT scoring results and quantitative radiological assessments at the time of US examination.

### 2.3. HRCT Assessment

HRCT was assessed by an expert radiologist.

All examinations were acquired in the supine position at full inspiration, without intravenous contrast. The protocol included:

- Volumetric acquisition with thin-section collimation (slice thickness 1–1.25 mm);
- A high-spatial-frequency reconstruction algorithm;
- A tube voltage of 100–120 kVp with dose modulation (low-dose HRCT protocol when feasible);
- Images reconstructed in the axial plane with multiplanar reformations when needed;
- Additional prone or expiratory scans performed only when clinically indicated (e.g., to differentiate dependent atelectasis or assess air trapping).

All HRCT scans included in the study were considered of diagnostic quality and were analyzed using the same reconstruction parameters, ensuring methodological consistency.

Semiquantitative scoring methods have been developed to provide more precise quantification of ILD abnormalities. One scoring system that has been used in several studies was developed and published by Warrick et al. [25]. This semiquantitative scoring system combines severity and extent of disease. An increased score is attributed to different abnormalities from 1 to 5: ground-glass opacities, irregular pleural margins, septal or subpleural lines, honeycombing and subpleural cysts corresponding to increasingly severe disease. Extent is determined based on the total number of bronchopulmonary segments involved for each abnormality and ranges from 1 (from 1 to 3 segments) to 3 (more than 9 segments involved). These scores are combined to obtain a global score that ranges from 0 to 30. In Table 2 we have detailed the various stages of the HRCT scoring procedure.

The study was approved by the local ethical committee of Area Vasta Nord Ovest (Tuscany—Italy) and each patient signed an informed consent form for participation in the study.

### 2.4. Statistical Analysis

All results were expressed in terms of mean value ± standard deviation (SD). The chi-square test, *t*-test and ANOVA were used to evaluate any differences in the subgroups analyzed, while ROC curves were used to evaluate the diagnostic capacity of the tests and to identify potential cut-offs. The correlation between continuous variables was analyzed by Pearson’s correlation. Inter-operator and intra-operator concordance was analyzed by Cohen’s kappa. Values of *p*-value < 0.05 were considered as statistically significant. All calculations were made using the SPSS Statistics 21.0 program.

## 3. Results

### 3.1. Disease Characteristics and Pulmonary Data

Fifty-three patients (36 females and 17 males) were enrolled and subgrouped as follows: 25 polymyositis (PM), 26 dermatomyositis (DM), and 2 inclusion body myositis (IBM). Mean age at enrollment was 62.9 ± 13.0 years and mean disease duration 49.8 ± 81.0 months. The data from disease activity parameters are reported in Table 2.

MSAs were positive in 26 patients (49%): Jo-1 (14 patients—26%), Pl-7 (5 patients—9%), SRP (2 patients—3%), TIF1gamma (5 patients—9%), Mi2 (1 patients—1.8%), MDA5 (1 patient—1.8%), and NXP-2 (1 patient—1.8%). Three patients were positive for two MSAs.

Disease activity parameters (according to International Myositis Assessment and Clinical Studies –IMACs-) of the analyzed patients are reported in Table 3.

Of the 53 examined patients, 8 (15%) were smokers at the time of enrolment in the study and 45 (85%) were non-smokers; of the latter, 20 (37%) were former smokers while 25 (47%) had never smoked. The average consumption of cigarettes, quantified in Pack-years, was 12.3 ± 7.2 for current smokers and 18.2 ± 17.1 for former smokers.

**Figure 1:**
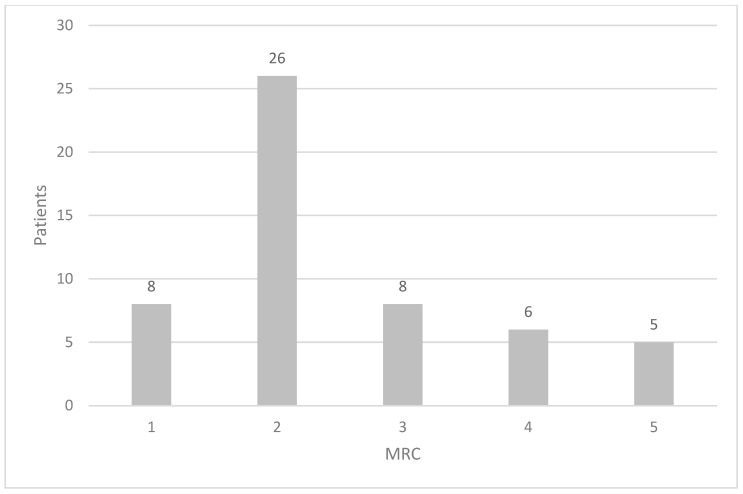
Grades of dyspnoea in the cohort of IIM according to the medical research council (MRC) scale.

Regarding respiratory symptoms, 8/53 patients (15.1%) did not report dyspnoea, while 45 patients presented different grades of dyspnoea according to the MRC scale (Figure 1).

Only 7 patients reported chronic cough symptoms (13.2%) and in 16 patients (31.4%), physical examination of the chest documented the presence of added respiratory noises, in particular fine crackles.

Pulmonary function test results are reported in Table 4.

The questionnaires for respiratory symptoms showed mean scores of 26.8 ± 17.2 for the SGRQ and 18.8 ± 3.3 for the LCQ.

### 3.2. Imaging Data

ILD was identified on HRCT in 37 out of 53 patients (69.8%). The mean Warrick score (WS) in the group with ILD was 7.5 ± 8.3, while a mean WS of 0 was identified in patients without ILD as by definition.

Lung US result agreement for the two operators was excellent (with an intra- and inter-operator agreement evaluated by Cohen’s kappa of, respectively, 0.944 and 0.901).

The mean sum of PIs for the comprehensive examination, identified by lung US, was 13.0 ± 9.6 for anterior spaces and 21.9 ± 13.9 for posterior spaces. The mean total PI score was 34.9 ± 22.1. As these three scores were strictly correlated with ρ > 0.758 and *p* < 0.001, for the further analyses we used only the total PI score.

The mean total PIs was not different in the disease subgroups (PM, DM, and IBM). No correlations were found between the total PIs and the IMACS disease activity parameters. Actual and former smoke habits were not significantly associated with differences in the mean total PIs.

Patients with MSAs had significantly higher total PIs than patients without MSAs (44.7 ± 23.5 vs. 25.4 ± 15.9; *p* < 0.001), in particular the 19 patients with anti-synthetase antibody positivity (49.0 ± 23.4 vs. 28.9 ± 17.4; *p* < 0.001).

**Figure 2:**
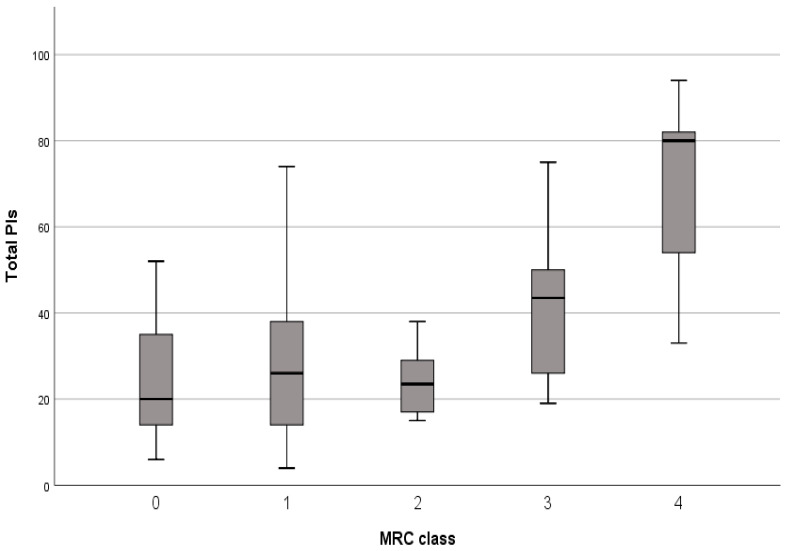
Trend of total PIs according to dyspnoea grade (medical research council—MRC).

The total mean PIs was not significantly different between patients with and without dyspnoea (35.9 ± 23.0 vs. 28.9 ± 15.2; *p* = n.s.) but an increasing trend for PIs according to the MRC grade was identified, especially in patients with MRC score 4, who presented significantly higher total PIs than patients with lower dyspnoea grades (*p* < 0.03) (Figure 2).

Higher PIs were identified in patients with chronic cough (52.5 ± 26.7 vs. 30.7 ± 18.3; *p* < 0.004) and in patients with fine crackles at the pulmonary clinical examination (46.0 ± 23.3 vs. 29.5 ± 18.6; *p* = 0.012).

**Figure 3:**
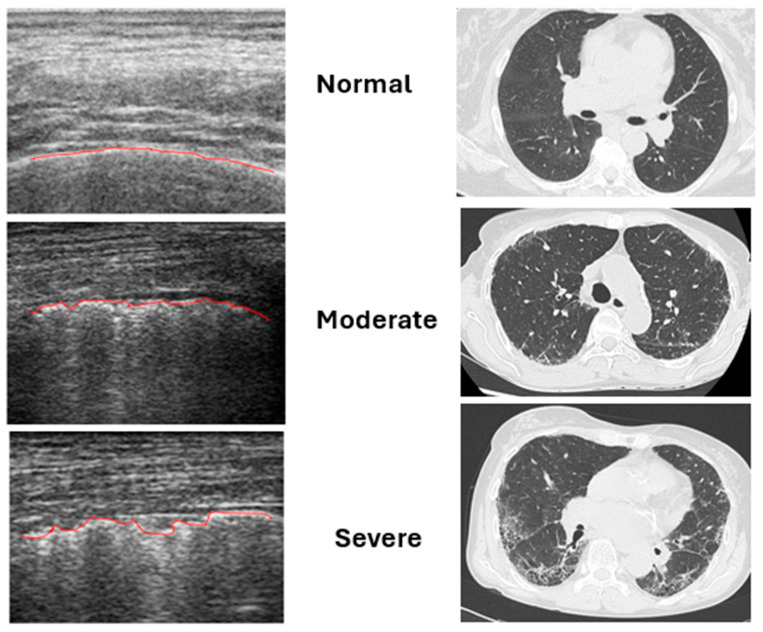
Correspondence between the severity of PIs and HRCT patterns. The red line represents the pleural line; its irregularities worsen in proportion to the severity of interstitial lung involvement.

The analysis of the spirometry data showed a strict negative correlation between total PIs and TLC (r = −0.74; *p* < 0.001) as well as between total PIs and DLCO (r = −0.56; *p* < 0.001). A negative correlation was identified between total PIs and the LCQ (r = −0.58; *p* = 0.001) while no correlations were found with the SRGQ. In patients without HRCT evidence of ILD, mild PIs detected by LUS were considered non-specific and possibly related to factors such as aging, minimal subclinical pleural changes, prior inflammatory or infectious events, and smoking-related pleural alterations. No patient without HRCT evidence of ILD showed extensive or diffuse severe PIs. Figure 3 illustrates the correspondence between the severity of PIs detected by LUS and the HRCT radiological patterns.

Patients with ILD identified with HRCT had higher total PIs compared with those without ILD (39.7 ± 22.9 vs. 22.8 ± 15.9; *p* = 0.015) and, in particular, there was a direct correlation between total PIs and Warrick score (r = 0.542; *p* < 0.001).

The average time required to perform lung ultrasound was:

- Approximately 9 min 53—intercostal space protocol;
- Approximately 3 min for the reduced 16—intercostal space protocol (postero-inferior regions only). Additional time may be required if findings are positive, particularly when distinguishing between moderate and severe changes.

### 3.3. Specificity and Sensitivity of Lung US

Specificity and sensitivity of lung US were evaluated according to the total number of PIs compared to the presence of ILD with HRCT. In the comprehensive scanning protocol, total PIs reached the maximum specificity when the score was higher than 27.5 (sensitivity 70% and specificity 81.3%), while the highest sensitivity was achieved with values higher than 17.5 (sensitivity 92% and specificity 37.5%). In the reduced scanning protocol, total PIs reached the maximum specificity when the score was higher than 12.5 (sensitivity 62% and specificity 95%), while the highest sensitivity was achieved with values higher than 6.5 (sensitivity 90% and specificity 40%).

Positive and negative predictive values (PPVs and NPVs) were also evaluated for both scanning protocols (Table 5).

## 4. Discussion

ILD is common in patients with IIM and it seems to affect females [26] and black people [27,28] more frequently. Although HRCT remains the gold standard for the study of ILD in patients with IIM, lung US may represent a fast, easy-to-perform, safe and reliable bedside tool for the evaluation of ILD. It is important to note that, to date, LUS exhibits limited efficacy in differentiating between various ILD radiological patterns. However, Rotondo et al. recently suggested that PIs are more prevalent than BLs as a distinctive marker for UIP [29]. Our results show that PI assessment may highlight ILD in the large majority of patients with IIM and seems to correlate with respiratory signs and symptoms. In particular, the total PIs was higher in patients with higher MRC dyspnoea grade, chronic cough and thoracic crackles at the pulmonary examination. Moreover, the total PIs was negatively correlated with TLC and DLCO and there was a good correlation between total PIs and the Leicester Cough Questionnaire’s score. By identifying a correlation between PIs, clinical symptoms and functional data, LUS could facilitate a symptom-driven, personalized assessment of ILD burden.

On the contrary, PI number did not significantly differ between previous and current smokers.

In agreement with the literature, anti-synthetase autoantibodies were confirmed as a significant risk factor for the presence of ILD in IIM patients [1,30,31]. This finding underscores the potential of LUS to refine risk stratification in IIM.

It is also interesting to note that there was a direct correlation between total PIs and Warrick semiquantitative score computed with HRCT, suggesting lung US might be used both as a screening and follow-up tool for patients with IIM. Finally, the results of lung US showed good intra- and inter-operator reliability. A cut-off of total PIs of 17.5 might be proposed as a screening test value to detect those patients who should undergo HRCT. Our data showed that PIs from anterior and posterior thorax fields are strictly correlated with each other and with the total PIs. This aspect of a relatively homogeneous distribution of ILD in patients with IIM was similarly observed in patients with CTDs in comparison with idiopathic ILD [32]. This observation, already proposed for the analysis of B-lines, might motivate an ultrasonographic evaluation of a reduced number of intercostal spaces, thus permitting a less time-consuming examination [33,34]. Interestingly, the reduced scanning protocol showed higher PPVs and NPVs than the comprehensive one. Therefore, a simpler examination might be suggested for routine use in clinical practice, to better select those patients for whom HRCT should be performed. This aspect is even more intriguing when the results of PFTs may be unreliable for respiratory muscle weakness, a common condition in patients with IIM. The observation of a significant difference in total PIs only in patients with severe dyspnoea, although a trend may be also identified for lower MRC classes, may suggest that patients with ILD may present clinically relevant symptoms only when the extent of the lung disease reaches a critical point. Therefore, it could be suggested the treating clinician should perform lung US even in asymptomatic patients, thus applying an easily performed and unexpensive screening assessment.

It is worth noting that our results are derived from an outpatient population. While LUS is a valuable bedside tool for routine monitoring, its performance and diagnostic accuracy might differ in acute clinical scenarios, which were not addressed in this study.

As shown in Table 6, our findings align with previous studies, confirming a strong correlation between LUS and HRCT findings. In particular, similarly to Pinal-Fernandez and colleagues, we analyzed the development of PIs. Furthermore, our study identified a correlation with PFTs and the Warrick score, thereby highlighting the relation among respiratory dysfunction, HRCT imaging, and the detection of PIs.

Although US evaluation is frequently considered as an operator-dependent imaging technique, our results confirmed that well-trained sonographers may ensure very good intra- and inter-operator reliability. Lung US has been proposed for the study of lung involvement in CTDs (in particular for the study of B-lines in patients with systemic sclerosis) and promising data have been published about the correlation with HRCT patterns [34,35,36]. Lung US has also already been proposed for patients with IIM [36]. Additionally, the analysis of PIs has been proposed as an alternative way to assess ILD, as it is easier to learn and quicker to perform than the analysis of B-Lines [15,37]. As already demonstrated by Pinal-Fernandez et al. [15], PIs showed a good correlation with the Warrick score. The prevalence of ILD in our cohort is lower (75%) than already published (90%) [15], thus reinforcing our hypothesis of the possible use of lung US as a screening tool. Moreover, our data refer to all IIMs and not only to patients with ASyS.

This work represents the largest cohort of IIM patients examined at the same time with both lung US and HRCT. The number of patients included is quite high considering the rarity of the disease. The main limitation of our work is that LUS might be less accurate in the examination of overweight patients or those with particular thoracic conformations. Another limitation of our study is the absence of a formal control group, specifically comprising IIM patients without ILD or individuals with other pulmonary disorders. While such a comparative analysis falls outside the scope of the current investigation, this evaluation is planned for future studies to further validate and strengthen our preliminary findings.

Moreover, our study did not include a control group of patients with other systemic autoimmune disorders. However, the existing literature provides relevant comparative data with both healthy and pathological cohorts. For instance, in patients with Spondyloarthritides, we recently observed a significantly higher prevalence of PIs compared to healthy subjects [16]. Furthermore, previous research comparing SSc patients with and without ILD demonstrated that PIs were exclusively present in those with confirmed pulmonary involvement [24]. These findings support the specificity of PIs as a marker for interstitial disease.

At present, the use of HRCT still remains an irreplaceable tool to analyze lung parenchyma and to guide ILD treatment. However, our results highlight the clinical utility of LUS in screening IIM patients for ILD. Furthermore, LUS allows for more frequent follow-up assessments than HRCT, given its superior safety profile and lack of radiation exposure. Nevertheless, further studies, ideally involving multicenter cohorts, are required to validate these findings.

Finally, we also demonstrated that the reduced postero-inferior scanning protocol showed comparable diagnostic performance with shorter examination time and, in our cohort, even higher PPVs and specificity than the comprehensive protocol. This likely reflects the known basal and posterior predominance of IIM-associated ILD. The focused approach therefore increases feasibility, reduces operator burden, and improves applicability in routine care without meaningful diagnostic loss. Moreover, the use of the reduced scanning protocol further personalizes the examination, focusing on the areas most likely to be affected in this specific patient population.

The comprehensive protocol remains valuable for research purposes and detailed mapping but may not be necessary for clinical screening or follow-up.

Based on these considerations, we propose a pragmatic algorithm for the application of LUS in the clinical management of ILD in patients with IIMs, with a focus on personalized medicine (see Table 7).

## 5. Conclusions

LUS represents a promising diagnostic tool for the bedside evaluation of patients with IIM; indeed, the study of PIs is a reproducible, rapid, and clinically significant marker of ILD burden in patients with IIM and could complete the clinical evaluation of lung involvement. Moreover, it could be easily performed, even in an outpatient setting, and our findings suggest that a focused posterior scanning protocol could be sufficient for routine clinical use. While LUS is not intended to replace HRCT, it serves as a valuable bedside adjunct to enhance patient monitoring and guide diagnostic strategies. This approach might optimize the use of HRCT in ILD assessment, thereby reducing patient exposure to ionizing radiation. From this point of view, integrating LUS into routine practice represents a step toward precision rheumatology, allowing clinicians to individualize the frequency of HRCT scans based on real-time bedside findings. This approach could not only optimize resources but also prioritize patient safety and comfort, aligning with the core goals of personalized healthcare.

However, more data are needed to better clarify which specific diagnostic strategy should be adopted, to improve quality of care in this context.

## 6. Research Agenda

Based on the findings of our study, the future perspectives for the use of LUS in assessing ILD in patients with IIM could be as follows:

- Validate LUS as a true screening tool in unselected and early IIM populations;
- Define standardized scanning protocols and cut-offs;
- Compare pleural irregularities vs B-lines vs combined scoring;
- Evaluate longitudinal change and treatment response prediction;
- Investigate machine learning or automated ultrasound quantification;
- Correlate ultrasound findings with specific HRCT patterns and histopathology;
- Investigate the role of LUS in personalized therapeutic algorithms to determine if PI changes can predict individual treatment response.

## Figures and Tables

**Table 1:** Criteria routinely used in our center for prescribing HRCT.

Persistent cough for at least 8 weeks
Dyspnoea
Detection of crackles at the chest physical examination
Signs of interstitial disease on chest X-ray
Spirometry data as total lung capacity < 80% and predicted reduction in the DLCO < 80%
Positivity of the anti-synthase antibodies (Jo-1, Pl-7, Pl-12, Oj, and Ej)

**Table 2:** The steps involved in the HRCT scoring process.

Step	Parameter	Criteria	Score
Step 1—Severity score (identification of ILD abnormalities)	Ground-glass opacities	Presence of the most severe abnormality	1 point
	Irregular pleural margins	Presence	2 points
	Septal/subpleural lines	Presence	3 points
	Honeycombing	Presence	4 points
	Subpleural cysts	Presence	5 points
	Note	Each abnormality is scored once if present, regardless of the number of lung segments involved	—
Step 2—Extent score (anatomical distribution)	Extent of each abnormality	1–3 bronchopulmonary segments involved	1 point
		4–9 segments involved	2 points
		>9 segments involved	3 points
Step 3—Total score calculation	Score for each abnormality	Severity score + Extent score	—
	Global Warrick score	Sum of all abnormality-specific scores	0–30
	Interpretation	Higher scores indicate more severe and extensive ILD	—

**Table 3:** Disease activity parameters according to IMACS.

	Parameter	Value (Mean ± SD)
Laboratory	Creatine kinase	985.3 ± 2610.3 IU/L
	Lactate dehydrogenase	312.4 ± 205.3 IU/L
Clinical parameters	Manual muscle test 8	67.3 ± 10.9 (0–80)
	Physician activity VAS	7.8 ± 13.9 (0–10)
	Patient’s activity VAS	4.4 ± 2.4 (0–10)
	MYOACT	16.9 ± 14.4 (0–100)
	Health assessment questionnaire	1.2 ± 1.1 (0–3)

Legend: IMACS: International Myositis Assessment and Clinical Studies, VAS: visual analog scale, and MYOACT: myositis disease activity assessment tool.

**Table 4:** Results of pulmonary function tests in IIM patients.

	Parameter	Value (Mean ± SD)
Spirometry	FEV1	98.9 ± 14.3%
	FEV1/VC	100 ± 12.3%
	TLC	88.4 ± 12.8%
DLCO	DLCO	75.3 ± 21.0%
	DLCO/VA	88.3 ± 22.8%

Legend: FEV1: forced expiratory volume at first second, VC: vital capacity, TLC: total lung capacity, DLCO: diffusing capacity of the lungs for carbon monoxide, AV: alveolar volume, and SD: standard deviation.

**Table 5:** PPVs and NPVs for both scanning protocols.

	PI Cut-Off	PPV	NPV	Accuracy
Comprehensive scanning protocol	27.5	90%	48%	69%
	17.5	77%	62%	74%
Reduced scanning protocol	12.5	96%	54%	72%
	6.5	78%	67%	75%

**Table 6:** A comparison of studies analyzing ILD via LUS in patients with CTDs.

Study	Disease	US Marker	Main Finding	Correlation with HRCT	Clinical Role
[15]	SSc/ASyS	Pleural irregularities	Correlate with fibrosis	Yes	Screening/monitoring
[35]	SSc	B-lines	Reflect interstitial involvement	Yes	Screening
[36]	IIM-ILD	B-lines	Correlate with ILD severity	Yes	Severity marker
Carli et al. (present study)	IIM (full spectrum)	Pleural irregularities	Correlate with DLCO, TLC, Warrick	Yes	Monitoring/HRCT guidance

**Table 7:** A personalized diagnostic algorithm for ILD management in IIM patients.

1	At IIM diagnosis: baseline HRCT + PFTs + LUS
2	Routine follow-up: periodic clinical assessment + PFTs + focused LUS
3	If LUS normal and stable PFTs: continue monitoring, defer HRCT
4	If LUS worsens, PIs are above threshold, or PFT decline: perform HRCT
5	If respiratory symptoms or acute deterioration: immediate HRCT regardless of LUS

## Data Availability

The data presented in this study are available on reasonable request from the corresponding author.

## Abbreviations

The following abbreviations are used in this manuscript:

ACR	American College of Rheumatology
ANOVA	Analysis of Variance
ASyS	Anti-Synthetase Syndrome
AV/VA	Alveolar Volume
BL	B-Line
CK	Creatine Kinase
CTDs	Connective Tissue Diseases
DAD	Diffuse Alveolar Damage
DLCO	Diffusing Capacity of the Lungs for Carbon Monoxide
DM	Dermatomyositis
EC	Examiner/Operator Initials
EULAR	European Alliance of Associations for Rheumatology
FEV1	Forced Expiratory Volume in the First Second
HAQ	Health Assessment Questionnaire
HRCT	High-Resolution Computed Tomography
IBM	Inclusion Body Myositis
IIM/IIMs	Idiopathic Inflammatory Myopathies
ILD	Interstitial Lung Disease
IMACS	International Myositis Assessment and Clinical Studies
LCQ	Leicester Cough Questionnaire
LDH	Lactate Dehydrogenase
LUS/Lung US	Lung Ultrasound
MAA	Myositis-Associated Autoantibody
MDA5	Melanoma Differentiation-Associated Protein 5
MMT8	Manual Muscle Test (8 Muscles)
MRC	Medical Research Council
MSA	Myositis-Specific Autoantibody
MYOACT	Myositis Disease Activity Assessment Tool
NPV	Negative Predictive Value
NSIP	Non-Specific Interstitial Pneumonia
NXP-2	Nuclear Matrix Protein 2
OMERACT	Outcome Measures in Rheumatology
OP	Organizing Pneumonia
PFTs	Pulmonary Function Tests
PIs	Pleural Irregularities
PM	Polymyositis
PPV	Positive Predictive Value
PRO	Patient Reported Outcome
RA	Rheumatoid Arthritis
ROC	Receiver Operating Characteristic
SB	Second Operator Initials(iniziali)
SD	Standard Deviation
SGRQ	Saint George Respiratory Questionnaire
SLE	Systemic Lupus Erythematosus
SSc	Systemic Sclerosis
SRP	Signal Recognition Particle
TIF1γ	Transcription Intermediary Factor 1-gamma
TLC	Total Lung Capacity
UIP	Usual Interstitial Pneumonia
US	Ultrasound
VAS	Visual Analog Scale
VC	Vital Capacity
WS	Warrick Score
